# Scale-up from microtiter plate to laboratory fermenter: evaluation by online monitoring techniques of growth and protein expression in *Escherichia coli *and *Hansenula polymorpha *fermentations

**DOI:** 10.1186/1475-2859-8-68

**Published:** 2009-12-22

**Authors:** Frank Kensy, Christoph Engelbrecht, Jochen Büchs

**Affiliations:** 1 AVT-Biochemical Engineering, RWTH Aachen University, Sammelbau Biologie, Worringerweg 1, 52074 Aachen, Germany; 2 m2p-labs GmbH, Forckenbeckstraße 6, 52074 Aachen, Germany

## Abstract

**Background:**

In the past decade, an enormous number of new bioprocesses have evolved in the biotechnology industry. These bioprocesses have to be developed fast and at a maximum productivity. Up to now, only few microbioreactors were developed to fulfill these demands and to facilitate sample processing. One predominant reaction platform is the shaken microtiter plate (MTP), which provides high-throughput at minimal expenses in time, money and work effort. By taking advantage of this simple and efficient microbioreactor array, a new online monitoring technique for biomass and fluorescence, called BioLector, has been recently developed. The combination of high-throughput and high information content makes the BioLector a very powerful tool in bioprocess development. Nevertheless, the scalabilty of results from the micro-scale to laboratory or even larger scales is very important for short development times. Therefore, engineering parameters regarding the reactor design and its operation conditions play an important role even on a micro-scale. In order to evaluate the scale-up from a microtiter plate scale (200 μL) to a stirred tank fermenter scale (1.4 L), two standard microbial expression systems, *Escherichia coli *and *Hansenula polymorpha*, were fermented in parallel at both scales and compared with regard to the biomass and protein formation.

**Results:**

Volumetric mass transfer coefficients (k_L_a) ranging from 100 to 350 1/h were obtained in 96-well microtiter plates. Even with a suboptimal mass transfer condition in the microtiter plate compared to the stirred tank fermenter (k_L_a = 370-600 1/h), identical growth and protein expression kinetics were attained in bacteria and yeast fermentations. The bioprocess kinetics were evaluated by optical online measurements of biomass and protein concentrations exhibiting the same fermentation times and maximum signal deviations below 10% between the scales. In the experiments, the widely applied green fluorescent protein (*GFP*) served as an online reporter of protein expression for both strains.

**Conclusions:**

The successful 7000-fold scale-up from a shaken microtiter plate to a stirred tank fermenter was demonstrated in parallel fermentations for standard microbial expression systems. This confirms that the very economical and time efficient platform of microtiter plates can be very easily scaled up to larger stirred tank fermenters under defined engineering conditions. New online monitoring techniques for microtiter plates, such as the BioLector, provide even more real-time kinetic data from fermentations than ever before and at an affordable price. This paves the way for a better understanding of the bioprocess and a more rational process design.

## Background

Industrial bioprocesses mainly involve microbial or mammalian fermentations. To develop a productive bioprocess, it is necessary to screen a vast number of different clones and media. Moreover, for industry, it is crucial to develop the bioprocess as fast as possible to reduce time to market. Meanwhile, companies are pressured by regulatory authorities (e.g. FDA, EMEA) to substantiate the actual knowledge of the process applied [1,2]. The Process Analytical Technology Initiative (PAT) by the FDA is one of the major programs to improve process understanding, and, therefore, the quality of pharmaceutical products. Consequently, in recent years high-throughput technologies have become well-established in research laboratories to perform high-throughput experimentation and to gain more insights into bioprocesses.

Microtiter plates (MTPs) play the predominant role as the microbioreactor of choice for high-throughput screening applications. They are applied for drug discovery, cell cultivation, enzymatic assays and immunoassays [3-7]. This microbioreactor platform is mainly used as a batch reactor, and the experimental results are very often simply evaluated by endpoint analysis. The application of MTPs under shaken conditions can further improve mixing and mass transfer conditions in cultivations. Recognizing the great potential of MTPs as a serious reaction platform, some groups have started to characterize engineering parameters in these formats. Besides the mixing time, the oxygen transfer capacity (OTR_max_) and the volumetric mass transfer coefficient (k_L_a) are generally characterized [4,8-11]. In order to better exploit the results of MTP experiments, it is important to precisely ascertain the scalability of MTPs to standard laboratory fermenters. Only if the microbioreactor performs identically to laboratory stirred tank fermenters (STF), it is possible to scale-up the data generated in high-throughput screenings and, thus, speed up the development timeline.

Micheletti et al. [12] and Islam et al. [13] compared microbial and mammalian fermentations as well as biotransformations in MTPs with those in a STF. The k_L_a-value hereby emerged as one of the key scale-up factors. Using matched k_L_a-values, they obtained comparable results at both the MTP and STF scale. Their research could be considered as the starting point of the scale-up from MTP to STF fermentations. This research, however, was based on laborious and error-prone sampling methods from the different reactor scales. In general, MTPs are recognized as being inexpensive, highly standardized and easy to handle. Nonetheless, MTPs would be an ideal platform for all tasks in fermentation science and bioprocess development if sampling and process data acquisition were easier to automate. Thus, Samorski et al. [14] introduced a new online monitoring technique for continuously shaken microtiter plates which was further advanced and validated by Kensy et al. [15]. This technique, called BioLector, was able to resolve this lack of online information from MTPs and facilitated the processing of high-throughput fermentations; it now provides all relevant fermentation parameters online.

The aim of this study is to elaborate a scale-up methodology from microtiter plate to stirred tank fermenter. The validation of the performed microbial fermentations was based on defined mass transfer conditions and online monitoring signals of biomass and protein concentrations at both reactor scales. Parallel fermentations of standard microbial expression systems such as the bacteria *Escherichia coli *and the yeast *Hansenula polymorpha *were performed in MTP with 200 μL and STF with 1.4 L scale and compared with each other.

## Methods

### Microorganisms and Media

Standard microbial expressions systems, the bacteria *Escherichia coli *and the yeast *Hansenula polymorpha*, were used for the scale-up experiments. Moreover, the widely used green fluorescent protein (*GFP*) was chosen as a model protein for protein expression, because it can be monitored online [16]. The strain *E. coli BL21(DE3) pRSET B GFP-S65t *which expresses *GFP *was kindly delivered by Markus Sack from Fraunhofer IME, Germany. The *E. coli GFP *expression was controlled by the strong, inducible *T7 *promoter (inducer: isopropyl-β-D-thiogalactopyranosid - IPTG). The GFP-S65T mutant processes a point mutation at position 65 from Serine to Threonine, which results in a single red shifted excitation peak, a more intensive fluorescence, and in an approximately four times faster fluorescence response than wild type GFP [17]. The strain *Hansenula polymorpha RB11-pC10-FMD-GFP *expresses *GFP *under the control of the *FMD *promoter [18]. The *FMD *promoter is repressed under the presence of glucose and derepressed under the presence of glycerol in the culture medium [18]. It was kindly provided by Carsten Amuel from the Institute of Microbiology, Heinrich-Heine-University Düsseldorf, Germany.

The *E. coli *experiments were carried out with the synthetic medium Wilms-Reuss (WR) [19]. The medium had the following composition: WR medium: 20 g/L glycerol, 2.0 g/L Na_2_SO_4_, 2.68 g/L (NH_4_)2SO_4_, 0.5 g/L NH_4_Cl, 14.6 g/L K_2_HPO_4_, 4.0 g/L Na_2_HPO_4 _× 2 H_2_O, 1.0 g/L (NH_4_)_2_-H-citrate, 0.5 g/L MgSO_4 _× 7 H_2_O, 0.01 g/L thiamine chloride hydrochloride, 3 ml/L trace element solution (TES), pH-value was adjusted to 7.2 with 1 M NaOH. TES contains: 0.5 g/L CaCl_2_, 0.18 g/L ZnSO_4 _× 7H_2_O, 0.1 g/L MnSO_4 _× H_2_O, 10.05 g/L Na_2_-EDTA, 8.35 g/L FeCl_3_, 0.16 g/L CuSO_4_, × 5H_2_O, and 0.18 g/L CoCl_2 _× 6H_2_O. The *E. coli *cultures were induced with 0.5 mM isopropyl-β-D-thiogalactopyranosid (IPTG, Biomol GmbH, Germany).

The *Hansenula polymorpha *experiments were carried out with the synthetic medium SYN6- MES, which had the following composition [20]: 20 g/L glycerol, 1.0 g/L KH_2_PO_4_, 7.66 g/L (NH_4_)2SO_4_, 3.3 g/L KCl, 3.0 g/L MgSO_4 _× 7H_2_O, 0.3 g/L NaCl, 27.3 g/L MES Pufferan (Carl Roth GmbH & Co. KG, Karlsruhe, Germany). After dissolution of all medium components, the pH-value was adjusted to 6.4 with 1 M NaOH or 1 M H_2_SO_4_. Then, the medium was autoclaved. After autoclaving of the basic medium, 6.67 mL/L micro element stock solution, 6.67 mL/L vitamin stock solution, 3.33 mL/L trace element stock solution (that have all been filter sterilized) and 6.67 mL/L calcium stock solution (that has been autoclaved) were added to the medium. The stock solutions had the following compositions: micro element stock solution: 10 g/L (NH_4_)_2_Fe(SO_4_)_2 _× 6 H_2_O, 0.8 g/L CuSO_4 _× 5 H_2_O, 3.0 g/L ZnSO_4 _× 7 H_2_O, 4.0 g/L MnSO_4 _× H_2_O, 10 g/L EDTA (Titriplex III, Merck, Darmstadt, Germany); vitamin stock solution: 60 mg/L D-Biotin, 20 g/L thiamine chloride hydrochloride; trace elements stock solution: 100 mg/L NiSO_4 _× 6 H_2_O, 100 mg/L CoC1_2 _× 6 H_2_O, 100 mg/L H_3_BO_3_, 100 mg/L KJ, 100 mg/L Na_2_MoO_4 _× 2H_2_O; calcium stock solution: 150 g/L CaCl_2 _× 2H_2_O.

The preculture for the fementations of both bacterial and yeast strains were prepared by using 5 cryo vials which were inoculated into 100 mL of the respective fermentation medium (WR for *E. coli *or Syn6-MES for *H. polymorpha*). Each inoculated culture volume (100 mL) was then distributed equally among five 250 mL Erlenmeyer shake flasks and was respectively incubated at a shaking frequency of 300 rpm, a shaking diameter of 50 mm, and a temperature of 30°C on an orbital shaker (LS-X, Kühner AG, Birsfelden, Switzerland). The starting optical density (OD_0_) of the *E. coli *preculture was 0.5, and that of the *H. polymorpha *preculture was 1.0. The precultivation of *H. polymorpha *took 18 hours and that of *E. coli *took 16 hours. After the preculturing, the five shake flasks were again pooled and 90 mL of the pool were inoculated into the fermenter resulting in a starting fermenter volume of 1.4 L after inoculation.

The precultures for the Respiration Activity Monitoring System (RAMOS) experiments were prepared by using only one cryo vial for inoculating 20 mL of the respective fermentation medium (WR for *E. coli *and Syn6-MES for *H. polymorpha*). The incubation conditions were the same as mentioned above.

All chemicals were of analytical grade and were delivered by Fluka/Sigma-Aldrich Chemie GmbH (Buchs, Switzerland) unless specified otherwise.

### Microtiter plate fermentation (BioLector)

**Figure 1:**
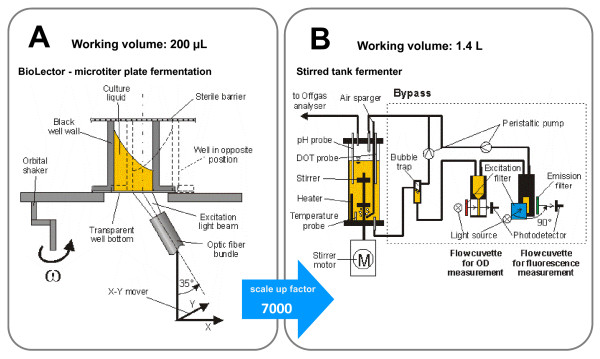
**Comparison of the experimental set up**. (**A**) Measurement principle of the microtiter plate fermentation in the BioLector via back scattering of light from cells and fluorescence emission of molecules; (**B**) Measurement principle of the stirred tank fermentation with online measurement of OD and fluorescence in the bypass.

The microtiter plate fermentations were conducted in the BioLector which was originally introduced by Samorski et al. [14] and recently improved and validated by Kensy et al. [15]. The general set-up of the BioLector is depicted in Figure 1A. The BioLector mainly consisted of an optical measurement unit (Fluostar, BMG Lab Technologies, Offenburg, Germany), an optical Y-fiber bundle (Prinz Optics GmbH, Stromberg, Germany), a X-Y mover (BMG Lab Technologies, Offenburg, Germany) and an orbital shaker (LS-X, Kühner AG, Basel, Switzerland). In the presented experiments the above described BioLector prototype was exclusively applied. The complete device has now been commercialized by m2p-labs GmbH, Aachen, Germany [21]. The biomass concentrations were measured via scattered light at 620 nm excitation without an emission filter. The *GFP *concentrations were monitored using an excitation filter of 485 nm and an emission filter of 520 nm. The sensitivity of the photomultiplier (gain) was adapted to the different measurement tasks and the detailed data is mentioned in the respective figures. The BioLector possessed a data reproducibility of below 5% standard deviation, upon cultivating the same clone in the same medium on a microtiter plate. Due to small standard deviation and the high information content, error bars in the figures were omitted. The experiments were exclusively carried out with black, standard round 96-well microtiter plates with an optical bottom from Greiner Bio-One GmbH (μClear, article number: 655087, Frickenhausen, Germany) that were covered with a gas permeable membrane from ABgene Ltd. (article number: AB-0718, Epsom, UK). If not otherwise specified, the experiments were conducted with 200 μL working volume of culture or medium, a shaking frequency of 995 rpm and a shaking diameter of 3 mm.

### Stirred tank fermenter

As standard stirred tank fermenter, a Visual-Safety-Fermenter (VSF, Bioengineering AG, Wald, Switzerland) with a nominal volume of 2 L was used. The fermenter possessed a standard *in-situ *dissolved oxygen tension (DOT) and pH electrode. The system was sterilized by autoclaving with an *in-situ *rod heater. The fermenter had a height-to-diameter ratio of 3 and two six-bladed Rushton turbines. The fermenter was aerated at a constant gas flow of 1 vvm controlled by a mass flow controller (5850TR, Brooks Instruments, Hatfield, PA, USA). The offgas from the fermenter was additionally analyzed by an online offgas analyzer (O_2 _by Magnos 106, ABB AG, Mannheim, Germany and CO_2 _by Unor 6N, Maihak AG, Hamburg, Germany). For the online measurement of OD and *GFP*, a liquid bypass was connected to the fermenter (Figure 1B). The fermentation broth was pumped from a bottom sample port of the fermenter by using a peristaltic pump (Fixo, Ismatec Laboratoriumstechnik GmbH, Wertheim-Mondfeld, Germany) through the bypass. This bypass contained a bubble trap (self-made) to avoid gas bubbles entering the optical detection line. For the OD detection, a flow cuvette with 0.5 mm path length (170-QS, Hellma GmbH& Co. KG, Müllheim, Germany) and a photometer (Photometer 6000, Skalar Analytical B.V., Breda, The Netherlands) were applied. The OD was measured with a 630 nm interference filter. Moreover, a flow cuvette (176.051-QS Hellma GmbH & Co. KG, Müllheim, Germany) and a fluorimeter (Fluorimeter 6300, Skalar Analytical B.V., Breda, The Netherlands) were applied for the *GFP *fluorescence measurements. The *GFP *was detected using an excitation filter of 480 nm and an emission filter of 530 nm. After passing through the flow cuvettes, the fermentation broth was recycled into the fermenter (Figure 1B). All online detected data were collected via a data acquisition module ADAM-4520 (Advantech Europe GmbH, Düsseldorf, Germany) and controlled by a self-developed bioprocess control program under LabVIEW (National Instruments Germany GmbH, München, Germany) on a PC.

The oxygen transfer rate (OTR) and carbon dioxide transfer rate (CTR) of the fermentation was calculated corresponding to the following equations (1) and (2), respectively [22,23]:

where y is the mole fraction of oxygen or carbon dioxide in the gas phase of the in or out flowing gas stream,  the norm gas flow rate and V_m _is the molar gas volume. From the online derived OTR measurement, the k_L_a-value of the fermenter could be determined as follows, assuming a completely mixed gas phase [22]:

where He_O2 _stands for the Henry constant for oxygen, p_R _for the reactor pressure, DOT for the dissolved oxygen tension and y_O2, cal _for the mole fraction of oxygen from the calibration gas of the DOT electrode. A Henry constant for oxygen of 1012 bar L/mol for the *H. polymorpha *fermentation and 993 bar L/mol for the *E. coli *fermentation was applied in the calculations (estimated from [24]).

The *E. coli *fermentation was conducted at a working volume of 1.4 L, a specific aeration rate of 1 vvm, a stirring speed of 1000 rpm and a temperature of 32°C. Unlike this fermentation, the *H. polymorpha *fermentation was operated at a working volume of 1.4 L, a specific aeration rate of 1 vvm, a stirring speed of 800 rpm and a temperature of 30°C. After the initial inoculation of the fermenter and a 5 min delay time for mixing of the cell suspension, a 10 mL sample was taken from the fermenter and was then distributed on the MTP to guarantee the same inoculation conditions. The same procedure was repeated with the *E. coli *fermentation after inducing the culture to have a second point of reference between the fermenter and the MTP.

### Respiration Activity Monitoring System

In the presented study, the Respiration Activity Monitoring System (RAMOS) was applied to determine the correlation between oxygen transfer rates (OTRs) obtained from the chemical sulfite system and biological cultures. RAMOS was first introduced and described in detail by Anderlei and Büchs [25]. Basically, the system consists of particular shake flasks, that in the lower part resemble equal to a standard Erlenmeyer shake flask and, in the headspace, contains an oxygen sensor in the gas phase to measure the respiration activity of cells. With this technique, the OTRs of the sulfite system, the *E. coli *fermentation in WR medium and the *H. polymorpha *fermentation in SYN6-MES medium (the same media later applied in the fermenter) were measured at different filling volumes of 10 mL, 15 mL and 20 mL at a shaking frequency of 250 rpm and a shaking diameter of 50 mm. The sulfite oxidation was performed at 25°C and the fermentations at 30°C. Under oxygen limiting conditions, the OTR curve of the sulfite oxidation and the fermentations forms a plateau. This plateau level of the different filling volumes can be correlated to each other, thereby giving a proportionality factor (f) between the OTRs of the biological media and the sulfite system as follows in equation (4) [11,26]:

The proportionality factor (f) represents the ratio between the different oxygen solubilities (C*) and diffusion coefficients (D_O2_) of the applied media.

### Determination of oxygen transfer rates (OTR) in surface-aerated bioreactors

For years, the sulfite oxidation served very well as a model system for the characterization of small-scale, surface-aerated bioreactors. Hermann et al. introduced this technique together with a simple color shift of a colorimetric pH-indicator making it possible to read out the reaction kinetics with a simple camera [9,10]. In the presented work, this sulfite oxidation method from Hermann was again applied to characterize the oxygen mass transfer conditions in 96-well microtiter plates with various filling volumes ranging vom 100 μL up to 260 μL. The 96-well microtiter plate was operated with a gas-permeable cover membrane (ABgene Ltd., article number: AB-0718, Epsom, UK) at a temperature of 25°C, a shaking frequency of 995 rpm and a shaking diameter of 3 mm.

### Calibration alignment between different optical measurement methods

**Figure 2:**
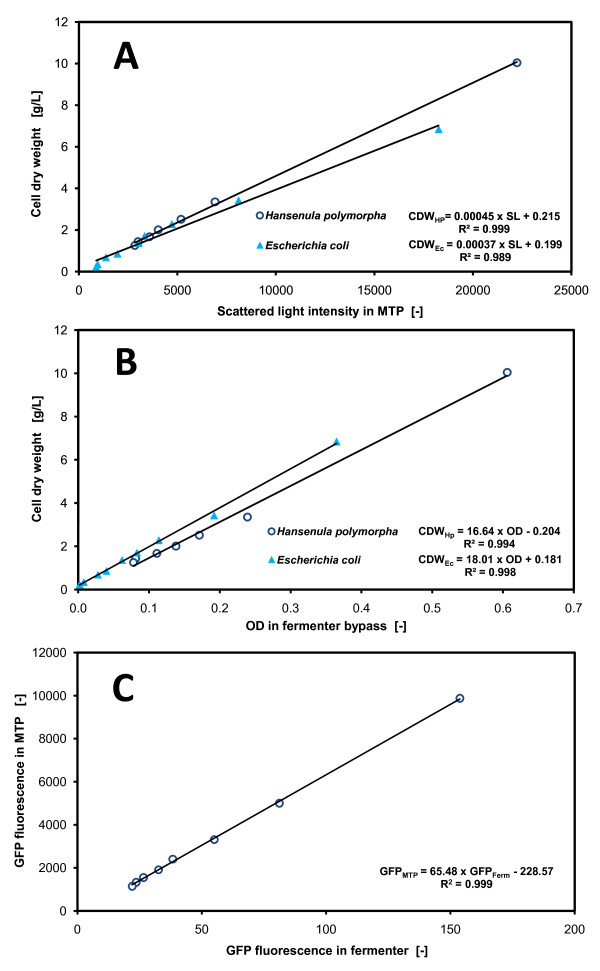
**Calibration of measurement signals**. (**A**) Cell dry weight versus scattered light intensities measured in microtiter plates; (**B**) Cell dry weight versus OD measured in the fermenter bypass; (**C**) *GFP *fluorescence in the microtiter plate versus *GFP *fluorescence in the fermenter of the *H. polymorpha *fermentation; Operation conditions: MTP (*H. polymorpha/E. coli*): 200 μL filling volume, at 30°C/32°C temperature, 995 rpm shaking frequency and 3 mm shaking diameter, scattered light (ex: 620 nm/em: -, gain: 20) and *GFP *(ex: 485 nm/em: 520 nm, gain: 10); fermenter (*H. polymorpha/E. coli*): 1.4 L filling volume, at 30°C/32°C temperature, 800/1000 rpm shaking frequency, 1 vvm specific aeration rate, OD (Absorption: 630 nm) and *GFP *(ex: 480 nm/em: 530 nm).

Due to different optical measurement methods applied in this study, the obtained measurement signals had to be calibrated with a standard analytical method. The scattered lights intensities from the BioLector and OD from the fermenter bypass were correlated to cell dry weight (CDW) as the biomass calibration unit. The cell dry weight was determined gravimetrically after washing the cells twice in physiological salt solution (9 g/L NaCl) and drying the cells at 105°C until the mass remained constant. The calibration of the measurement signals, i.e. scattered light and OD, with cell dry weight was conducted at the end of the fermentations. The contents of the fermenter analogous to the normal fermenter operation were recycled through the fermenter bypass. Simultaneously, the measurements, OD and *GFP *fluorescence, were analyzed. During the calibration procedure, the fermenter contents were continuously diluted with new medium, directed through the bypass and again measured. After each dilution step, a 5 mL sample was taken from the well mixed fermenter in order to collect scattered light intensities in the BioLector and to analyze cell dry weights from the same samples. This procedure was repeated for the fermentations of *E. coli *and *H. polymorpha*. Finally, these measurement signals could be correlated to each other (Figure 2A and 2B). Since no offline protein analysis was available in our laboratory for the detection of *GFP *concentrations, the *GFP *fluorescence signals of both measurement systems, the fermenter and the MTP, were directly correlated with each other (Figure 2C). From the literature, it is known that the *GFP *fluorescence signals can be well correlated with real *GFP *protein concentrations in well aerated systems such as fermenters [27].

## Results and Discussion

### Calibration of the measurement apparatus

In order to compare the growth and protein expression of both studied cultivation systems, i.e. the shaken microtiter plate and the stirred tank fermenter, it is very important to align the online measured parameters. Therefore, the biomass measurements of scattered light intensities in the MTP and those of OD in the STF bypass were all correlated to the cell dry weight. In the case of the protein expression parameter, *GFP *was applied as a model protein for simplifying the detection by fluorescence. *GFP *was not correlated to any offline laboratory method such as ELISA or Western Blot. Both the BioLector and the STF measurement systems were directly correlated to each other. Figure 2 presents the calibration of the measurement signals for the *E. coli *and *H. polymorpha *fermentations.

Figure 2 demonstrates that all correlations are highly precise. This is confirmed by the coefficient of determination value (R^2^) of approximately 0.99 which was calculated for all linear equations. Each R^2 ^is presented below the respective linear equations in Figure 2. For the measurement of scattered light in the MTP and of OD in the bypass, it was necessary to perform separate calibrations for the distinct microorganisms, *E. coli *and *H. polymorpha*, because the optical biomass measurement depends strongly on the morphology of the cells. For the *GFP *fluorescence measurements, however, it was only necessary to perform one calibration, because, in this case, it served only to align the different measurement systems.

The calibrations performed here were subsequently used to calculate the cell dry weight and the *GFP *fluorescence (based on the MTP fluorescence unit) for each fermentation. Thus, the kinetic data from both fermentation systems, MTP and STF, were actually comparable.

### Transformation of chemical OTR_max _to biological OTR_max_

**Figure 3:**
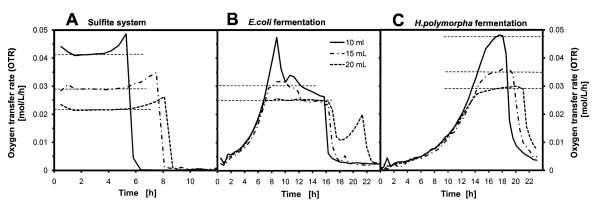
**Determination of the different oxygen transfer rates (OTR) in RAMOS**. (**A**) for the chemical 0.5 mM sulfite system; (**B**) for the *E. coli *fermentation in WR medium; (**C**) for the *H. polymorpha *fermentation in SYN6-MES medium. Operation conditions: 10 mL, 15 mL and 20 mL filling volume, 250 mL Erlenmeyer shake flask for RAMOS, 250 rpm shaking frequency, 50 mm shaking diameter, at 25°C temperature with the sulfite system and 30°C temperature with the microorganisms. The E. coli cultures were induced at 7.17 h with 0.5 mM IPTG.

The sulfite oxidation method is a very good and simple method to characterize the maximum oxygen transfer capacity (OTR_max_) in small-scale, surface-aerated fermentation systems as was reported by Hermann et al. [9]. Unfortunately, this chemical model system does not truly reflect the composition of biological media. In general, the actual medium composition interferes with the oxygen diffusion coefficient (D_O2_) and the oxygen concentration at the gas-liquid interface (C*). To determine the influence of the hereby applied fermentation media, additional RAMOS experiments with the sulfite system, the *E. coli *medium and *H. polymorpha *medium were necessary. Since no dissolved oxygen can be measured in the RAMOS device, it is important to run the experiments under oxygen-limiting conditions, where the oxygen concentration in the bulk liquid (C_L_) becomes very small. Consequently, the sulfite oxidation and the fermentation were run with different filling volumes (10 mL, 15 mL and 20 mL) in the RAMOS shake flasks. The results of this experiment are presented in Figure 3.

Figure 3 clearly depicts that most of the OTR curves possess a plateau, which implies the oxygen limitation [25,28]. Only the *E. coli *fermentation with 10 mL filling volume does not become limited and thus, forms a sharp peak. The graphs also depict that the sulfite system reached an OTR plateau directly after the start of the experiment, because the oxidation is driven by a constant concentration of the cobalt catalyst under almost constant reaction conditions [9]. Therefore, the reaction runs at an almost constant rate until the end of the experiment. In contrast, the fermentation cultures pass through the typical exponential growth phase until they reach the oxygen limitation and the mentioned OTR plateau.

In general, it is obvious that lower filling volumes attain higher OTR_max_-values than larger filling volumes due to the higher specific surface area assessable for oxygen diffusion [26,29]. For both microorganisms, i.e. *E. coli *and *H. polymorpha*, the maximum OTR necessary to supply sufficient oxygen to the culture ranges from 0.045-0.050 mol/L/h as is observed with the 10 mL filling volume. From these graphs the OTR plateau values for the different media were correlated to each other and inserted into Equation (4). Table 1 presents the evaluated proportionality factor (f):

**Table 1:** Evaluated proportionality factor (f) between fermentation and the sulfite system

**Proportionality factor (f)**	
*E. coli *in WR medium	**1.13**

*H. polymorpha *in SYN6-MES medium	**1.22**

These proportionality factors (f) were subsequently applied for calculating the maximum oxygen transfer capacities in MTPs.

### Characterization of k_L_a in MTPs

**Figure 4:**
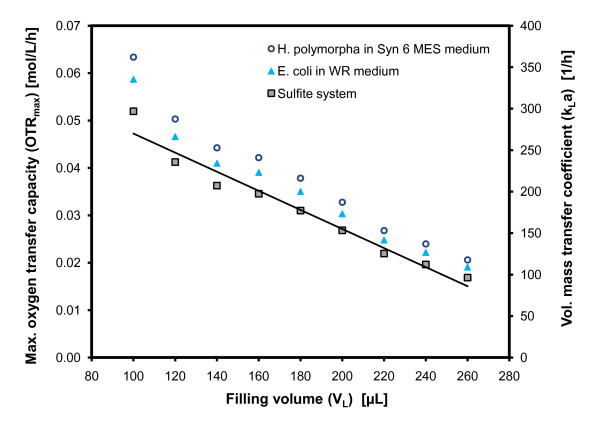
**Maximum oxygen transfer capacity (OTR_max_) and k_L_a-values versus the filling volume**. for the chemical 0.5 mM sulfite system, the *E. coli *fermentation in WR medium and the *Hansenula polymorpha *fermentation in SYN6-MES medium. The method from Hermann et al. [9,10] was applied for the determination and calculation of OTR_max _and k_L_a-values for the sulfite system. The OTR_max _and k_L_a-values for the fermentations were calculated by applying the proportionality factor (f) from table 1 on the OTR_max_-values of the sulfite system. Operation conditions (Sulfite system/*E. coli/H. polymorpha*): various filling volumes between 100-260 μL, at 25°C/30°C/30°C temperature, 995 rpm shaking frequency and 3 mm shaking diameter.

Upon cultivating microorganisms in small-scale bioreactors, it is very important to conduct the experiments under well-characterized operating conditions. To meet this requirement, the operation conditions in MTPs were first characterized using the sulfite oxidation method introduced by Hermann et al. [9]. Whereas, the shaking frequency and diameter were kept constant at 995 rpm and 3 mm, respectively, the filling volumes of the wells of the MTP were varied between 100 μL and 260 μL. The results of this characterization are presented in Figure 4.

It is notable that a linear correlation between the maximum oxygen transfer capacity (OTR_max_) and the filling volume was obtained, analogously as was reported previously [10,30,31]. The OTR_max _ranges from 0.017 mol/L/h (k_L_a = 97 1/h) at 260 μL up to 0.052 (k_L_a = 297 1/h) at 100 μL for the sulfite system. These values correlate very well to those reported from Hermann et al. [10] and John et al. [30] for standard round 96-well MTPs and also correlate well in magnitude with other formats such as square 24-deepwell plates [13]. Applying both aforementioned proportionality factors (f) for the fermentation media (Figure 4), the OTR_max _and k_L_a-values achievable in *E. coli *and *H. polymorpha *fermentations lie above the line of the sulfite system, whereby *H. polymorpha *always shows the highest values.

Even though, it would be better to apply filling volumes of 100-120 μL with regard to optimize the oxygen transfer (0.045-0.050 moL/L/h from Figure 3), an actual filling volume of 200 μL was applied in order to ensure that sufficient liquid was available for further offline analysis. Moreover, for a precise optical detection it was important to have enough liquid on the well bottom during the continuous shaking of the MTP [11,31]. This implies operating the MTP at a suboptimal k_L_a-value of 180-190 1/h compared to that of the stirred tank fermenter (k_L_a = 370-600 1/h). Nevertheless, the RAMOS experiments (Figure 3B and 3C) depicted that even oxygen-limiting OTRs of greater than 0.030 mol/L/h (k_L_a = 175 1/h) simply result in a prolongation of the fermentation by 1 h for *E. coli *and *H. polymorpha*, respectively. This is regarded as being non significant.

### Comparison of parallel *E. coli *fermentations in MTP and fermenter

**Figure 5:**
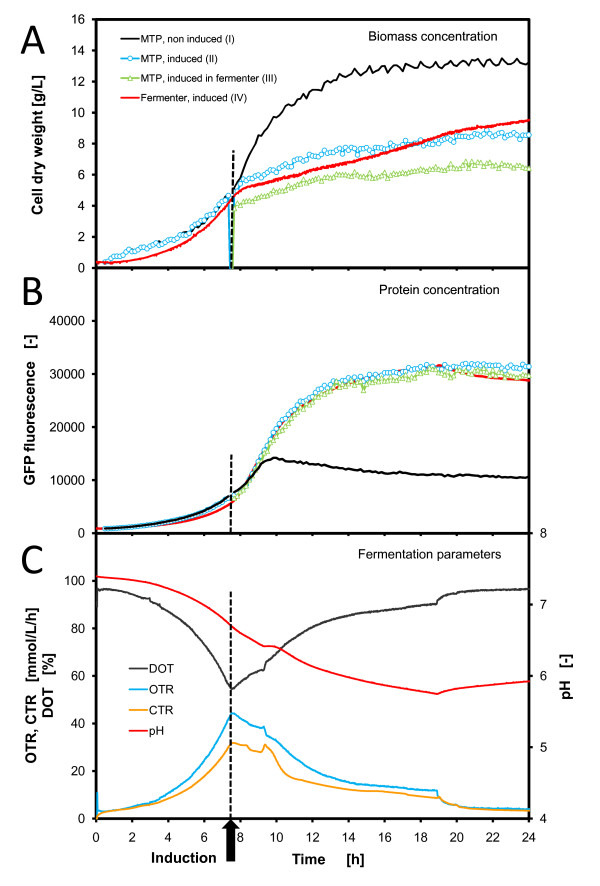
**Comparison of parallel *E. coli *fermentations in microtiter plate and stirred tank fermenter**. Operation conditions: MTP: 200 μL filling volume, at 32°C temperature, 995 rpm shaking frequency and 3 mm shaking diameter, scattered light (ex: 620 nm/em: -, gain: 20) and *GFP *(ex: 485 nm/em: 520 nm, gain: 10); fermenter: 1.4 L filling volume, at 32°C temperature, 1000 rpm shaking frequency, 1 vvm specific aeration rate, induction with 0.5 mM IPTG (end concentration in the fermenter) at 7.3 h, OD (Absorption: 630 nm) and *GFP *(ex: 480 nm/em: 530 nm)

Figure 5 presents the direct comparison of parallel *E. coli *fermentations in the MTP and STF.

Four different curves are plotted in Figure 5A and 5B:

• (I) MTP, non induced - the same *E. coli *fermentation without induction as a negative control

• (II) MTP, induced - the inoculated *E. coli *culture was taken from the fermenter and were distributed on the MTP, induction was performed at 7.3 h in the MTP

• (III) MTP, induced in fermenter - the *E. coli *culture was sampled from the fermenter after induction and then distributed on the MTP

• (IV) Fermenter, induced - the fermentation was completely running in the fermenter with an induction at 7.3 h.

Figure 5C presents the online process parameters from the fermenter and the offgas analyzer.

The development of the biomass concentrations (Figure 5A) depicts that, in the pre-induction phase, all three cultures (I, II and IV) have the same slope and reach a biomass concentration of 4.5 g/L at the induction point of 7.3 h (please note: the monitoring of the culture (III) - MTP, induced in fermenter - started after the induction, in the pre-induction phase this culture is displayed as the fermenter curve). In the post-induction phase, a clear separation of the negative control (I), which was not induced, is notable. All other cultures (II, III and IV), which were induced, follow the same trend. Obviously, the *E. coli *strain seems to be growth-inhibited after induction. Only at the very end of the fermentation, the fermenter curve (IV) deviates slightly from the MTP curves (II and III). This both induced cultures running in the MTP (II and III) show exactly the same growth behavior at a slightly deviating biomass concentration.

Analyzing the protein expression (Figure 5B) results in a very similar behavior. In the pre-induction phase, all three cultures (I, II and IV) follow the same slope resulting in a *GFP *fluorescence of 6500 at the induction point. It seems, that *E. coli *constitutively produces the product *GFP*. Then, after the induction, the curves of the different cultures deviate. The negative control (I) continues to produce *GFP *up to 10 h. Then, the *GFP *formation stagnates. In contrast, the induced cultures (II, III and IV) continue producing the *GFP *up to 19 h. All induced cultures (II, III and IV) follow exactly the same slope of *GFP *formation, which means that the cultivation conditions are indeed comparable for the MTP and the fermenter system. It cannot be excluded that GFP inclusion bodies have been formed in this expression experiment, but due to the steady slope of the GFP fluorescence signals it is expected that GFP is produced in soluble form.

Figure 5C exhibits the corresponding process parameters measured in the fermenter. All these parameters correspond very well to the different culture phases. Due to the missing availability of pH-control in the MTP, the fermenter was also operated without pH-control. This results in a pH decrease from 7.2 to 5.9 over the course of fermentation, which did not limit the culture growth. The OTR curve points out that in this fermentation, a maximum OTR of 0.043 mol/L/h was reached which is comparable to those values detected in shake flasks with RAMOS. k_L_a-values of 600 1/h were calculated with equation (3) for the STF which were almost three times higher than that applied in the MTP. The DOT curve in Figure 5C demonstrates that at this high k_L_a-value in the fermenter the *E. coli *culture reached a minimum DOT of 55%. This signifies that the culture is far away from an oxygen limitation. Even at the reduced k_L_a-value of the MTP (180 1/h), the culture experienced only a slight oxygen limitation which did not sustainably effect the culture growth nor protein expression as mentioned before. This minor influence of a slight oxygen limitation was also observed in the RAMOS experiments comparing the 10 mL and the 15 mL filling volumes (Figure 3B). In this case, a short prolongation of the fermentation with 15 mL filling volume in comparison to the 10 mL fermentation was noticeable.

Overall, the results of the parallel *E. coli *fermentations in MTP and STF demonstrate that the cultivation conditions are very comparable. All induced cultures behave very similarly and show the same growth and protein expression kinetics. The negative control of a non-induced culture depicts which differences in kinetics could normally appear.

### Comparison of parallel yeast fermentations in MTP and fermenter

**Figure 6:**
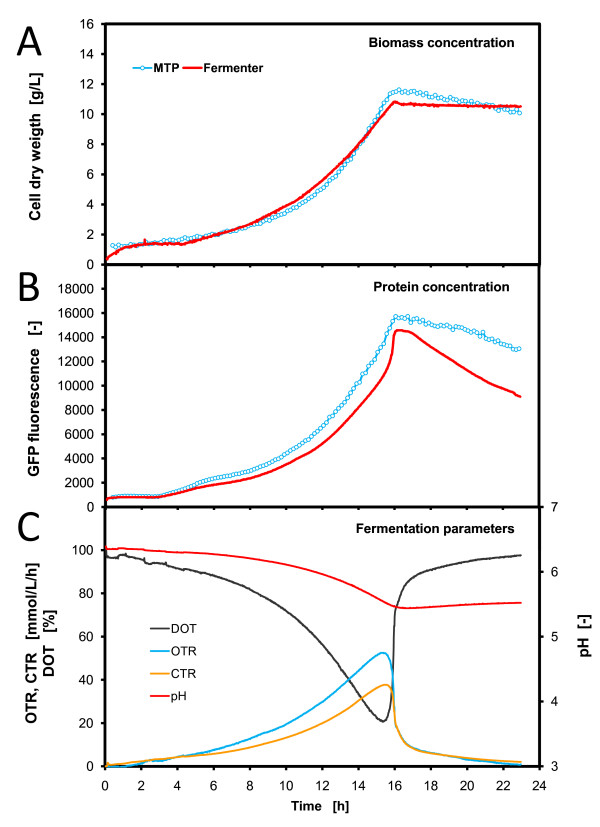
**Comparison of parallel *H. polymorpha *fermentations in microtiter plate and stirred tank fermenter**. Operation conditions: MTP: 200 μL filling volume, at 30°C temperature, 995 rpm shaking frequency and 3 mm shaking diameter, scattered light (ex: 620 nm/em: -, gain: 20) and *GFP *(ex: 485 nm/em: 520 nm, gain: 10); fermenter: 1.4 L filling volume, at 30°C temperature, 800 rpm shaking frequency, 1 vvm specific aeration rate, OD (Absorption: 630 nm) and *GFP *(ex: 480 nm/em: 530 nm).

The same experiment as for *E. coli *was also performed with the yeast *H. polymorpha*. Here, no induction of the promoter was necessary due to the different promoter regulation of *H. polymorpha*. The applied *FMD *promoter was simply derepressed by the substrate glycerol [18]. Very comparable growth and protein expression kinetics were also found in these parallel fermentations (Figure 6). In Figure 6A, for both cultivation systems, MTP and STF, almost identical exponential growth curves could be observed. Exactly at about 16 h, both fermentations enter the stationary phase at a biomass concentration of 11.4 g/L CDW (MTP) and 10.7 g/L CDW (STF) resulting in a relative error of 6.3%. Both systems also showed consistent protein expression of *GFP*. Even small changes in the expression rate appeared synchronously. In general, the protein expression mirrored the exponential growth curves. The protein formation was subjected to a derepression as known from the literature [18]. At the synchronous entry into the stationary phase at 16 h, both fermentations differ only by 9.5% with regard to the *GFP *fluorescence.

The process parameters of the fermenter (Figure 6C) again correspond very well to the biomass and protein formation kinetics. The OTR and CTR indicate an exponential development similar to the growth curve. All dynamics terminate at 16 h, when the substrate is exhausted and the metabolic activity decreases immediately. The uncontrolled pH-value decreases from 6.4 to 5.5, which does not limit growth of *H. polymorpha *[32]. A maximum OTR of 0.052 mol/L/h was reached, which is slightly higher than the values attained in the shake flasks in RAMOS (Figure 3C). The calculated k_L_a-value applied in the fermenter were 370 1/h which is double of that applied in the MTP (k_L_a = 180-190 1/h). In the fermentation the DOT dropped down to a minimum of 21% (Figure 6C). This might mean that the yeast culture in the MTP fermentation could run into a slight oxygen limitation. Again here, no significant influence is noticeable concerning culture growth and protein expression in comparison to the STF. Similar ratios of OTR between the 10 mL and 15 mL filling volumes in the RAMOS experiments (Figure 3C) as that applied in the STF and MTP only led to a minor prolongation of fermentation in RAMOS of 1 h. This could not be observed in the comparison of the STF and the MTP.

In summary, the MTP can mimic the fermenter very well. Besides the *E. coli *fermentations, the parallel *H. polymorpha *fermentations in the MTP and STF also produced very comparable growth and protein expression kinetics. During metabolic activity, all online-measured data show deviations of below 10% between the two cultivation systems.

### Characterization of specific product formation

**Figure 7:**
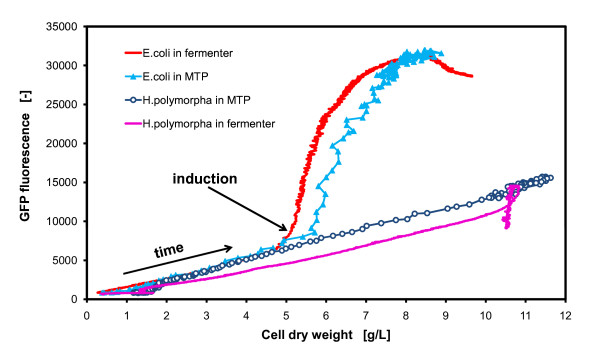
**Protein expression as function of cell dry weight - characteristics of different expression systems**. Direct correlation of the protein expression via *GFP *fluorescence to the biomass concentration of the fermentations of figures 5 and 6; calibrated data from measurements in the BioLector: scattered light (ex: 620 nm/em: -, gain: 20), *GFP *(ex: 485 nm/em: 520 nm, gain: 10) and in the fermenter bypass: OD (Absorption: 630 nm) and *GFP *(ex: 480 nm/em: 530 nm).

As it was demonstrated in the previous figures, fermentations in MTPs can mimic growth and protein expression kinetics very well. Therefore, it could be very interesting to apply the online measurement data of the MTP-based BioLector to characterize clones and fermentation conditions with respect to their specific product formation capacities. Figure 7 gives an example of a different presentation of the online data. With the BioLector, it is now possible to present the product formation data as a function of biomass concentration. This is rarely possible with fermenters, because laboratories often lack online biomass and fluorescence sensors. This online biomass measurement would be possible by applying an online OD measurement (like in our case in the bypass, Figure 1) or an online impedance sensor (e.g. from Aber Instruments, UK or Fogale, France). For the detection of fluorescent proteins an online fluorescence sensor could be installed (e.g. BioView from Delta, Denmark). These sensors, however, would require a much higher investment, because two additional sensors for each fermenter will be needed beside the common pH and DOT electrodes and the temperature probe.

Figure 7 presents the fluorescence development of *GFP *versus the cell dry weight of the *E. coli *and the *H. polymorpha *fermentation in both reactor scales, STF and MTP, which has been described earlier in this paper. Whereas, the *H. polymorpha *strain shows only a linear correlation between biomass and *GFP *protein, the *E. coli *strain experiences a boost in protein production after the induction (as indicated). The graphs in Figure 7 clearly depict the derepressed, almost constitutive, expression for the *H. polymorpha *strain and the induced expression for the *E. coli *strain. Additionally, the graphs in Figure 7 provide the specific product yield (Y_P/X_) of the strains by deriving the slope of the graphs as Y_P/X_. This evaluation criteria is very often used to consider the productivity of a fermentation process [2,33]. This novel way of data presentation, i.e. protein concentration as a function of biomass concentration, has first been made possible even on a micro-scale by using new online measurement techniques such as the BioLector. This can help to facilitate the selection of a potent clone or a very productive fermentation medium or process.

## Conclusions

After the validation of the online monitoring capacity of the new high-throughput fermentation system [15], called BioLector, it was challenging to investigate the scalability from a microtiter plate to a stirred tank fermenter. Therefore, the present study was focused on the scale-up of common microbial expression systems, such as the bacteria *E. coli *and the yeast *H. polymorpha*, from microtiter plate to stirred tank fermenter. The prerequisite to perform such a study was the comprehensive characterization of mass transfer conditions in microtiter plates. In recent years, many of the common microtiter plates were already characterized by several groups, whereby a few new methods for the characterization of engineering parameters were developed [9-11,29,34]. In this work, the maximum oxygen transfer capacity (OTR_max_) and the volumetric mass transfer coefficient (k_L_a) were determined with the sulfite oxidation method from Hermann et al. [9]. Specifically, the OTRmax and k_L_a were measured for various filling volumes in a standard 96-well microtiter plate at a constant shaking frequency of 995 rpm and a shaking diameter of 3 mm, because only little information was available at these conditions. Finally, it was necessary to convert the chemical OTR_max_-values into biological OTR_max_-values, which could be achieved with the help of a RAMOS device. Even though more practical but suboptimal filling volumes of 200 μL were applied instead of superior 100-120 μL with regard to the OTR-demand of the cultures, excellent scalability results could be attained. The comparison of the online measurement signals, biomass concentration and the *GFP *fluorescence as a model protein, in parallel fermentations using microtiter plate and stirred tank fermenter proved that the kinetics of growth and protein expression are well comparable between both reactor scales. This was confirmed not only by the bacteria fermentation of *E. coli*, but also by the yeast fermentation of *H. polymorpha*. Both expression systems showed nearly identical kinetics in the microtiter plate and the stirred tank fermenter, showing a maximum of 10% deviation between the measurement signals. Taking into account that the scale-up factor applied here was 7000, this was a surprisingly good result. Ultimately, the presented study pointed out that the scale-up from microtiter plates and stirred tank fermenters is possible for standard microbial expression systems with general low viscosity. As previously mentioned, one of the key factors for successful scale-up was the k_L_a-value [13]. This proven scalability of MTPs to STFs could make them ideally suited as a microbioreactor and a scale-down reactor unit. This scalability combined with high-throughput and online monitoring of important process parameters, i.e. biomass and protein concentrations when using fluorescent proteins or fusions thereof, creates a very powerful tool in screening and bioprocess development. Even though k_L_a- values of up to 350 1/h have been reported for round 96-well MTPs in this paper and for square 24-well MTPs in other publications [13], these k_L_a-values are still not sufficient to mimic a full industrial fermenter in a scale-down model. Higher k_L_a-values of up to 1000 1/h would be desirable to attain higher cell concentrations and to operate a fedbatch process. New microtiter plate formats with new geometrical well designs such as the recently presented Flowerplate could probably solve these limitations in the future [35].

## Competing interests

The authors declare that they have no competing interests.

## Authors' contributions

FK conceived the study and drafted the figures and the manuscript. CE performed all fermentations, calibrations and data analysis. JB participated in its design, coordination, and drafting of the manuscript. All authors read and approved the final manuscript.
